# Finite element analysis of restoring length with multiple internal fixations in calcaneal body fracture

**DOI:** 10.1038/s41598-024-75267-7

**Published:** 2024-10-10

**Authors:** Xiang Yao, Peiqi Ding, Chong Wang, Han Miao, Yicong Chao, Jiawei Wang, Minjie Hu, Jilei Tang

**Affiliations:** 1 Department of Orthopaedics, The Affiliated People’s Hospital of Jiangsu University, Zhenjiang, 212000 Jiangsu China; 2 Jiangsu University, Zhenjiang, 212000 Jiangsu China; 3 Department of Orthopaedics, The People’s Hospital of Danyang, Zhenjiang, 212300 Jiangsu China; 4 Department of Orthopaedics, Qidong Hospital of Traditional Chinese Medicine, Nantong, 226200 Jiangsu China

**Keywords:** Internal fixation, Calcaneal fracture, Shortening deformity, Locking plate, Finite element analysis, Distraction screw, Lag screw, Biomedical engineering, Medical research, Computer science

## Abstract

Calcaneal body fractures are often associated with varying degrees of shortening deformities. Restoring calcaneal length is crucial for the functional prognosis of the foot. Through finite element analysis, this study compared the biomechanical effects of multiple fixation schemes for calcaneal fractures. We delineated and assembled the finite element model of the Sanders type II calcaneal fracture and four internal fixation simulations (namely distraction screw, lag screw, frame locking plate, and T-shaped locking plate). Different axial forces (350, 700, and 1400 N) were then applied to simulate various postures. We then compared the inner and outer shortening distances (D1 and D2, respectively), equivalent von Mises stress, and maximum von Mises stress of the calcaneus. In the individual model, with an increase in the pressure, D1, D2, and the maximum von Mises stress gradually increased. At 1400 N, D1 and D2 for the internal fixation schemes were as follows: distraction screw (0.03 mm, 0.1 mm) < T-shaped locking plate (0.45 mm, 0.26 mm) < frame locking plate (0.50 mm, 0.26 mm) < lag screw (0.66 mm, 0.64 mm). The maximum von Mises stress values for the internal fixation methods were as follows: lag screw (491.0 MPa) < distraction screw (663.1 MPa) < frame locking plate (772.7 MPa) < T-shaped locking plate (931.8 MPa). In patients with calcaneal body fractures, the distraction screw is a potential therapeutic option for resisting calcaneal shortening.

## Introduction

Calcaneal fractures, the most common tarsal bone fracture, lead to approximately 75% of foot fractures and 1%–2% of all fractures^1^. They usually manifest as shortening of calcaneal length, narrowing of calcaneal width, and different degrees of articular surface injury. Among them, the deformity of calcaneal shortening is the most crucial pathological change in severe fractures^2^, with excessive calcaneal shortening causing dysfunctional gait and requiring calcaneal lengthening surgery, osteotomy, or subtalar arthrodesis as treatment^3–5^.

To reconstruct a healthy lower extremity, a calcaneal operation is primarily focused on restoring the calcaneal length by using various traction instruments (reduction forceps, and unilateral or bilateral distractor)^6^. This is regardless of whether open reduction, minimally invasive, or percutaneous internal fixation is used^7–11^. Determining how to fix distracted bone fragments so as to maintain the physiological calcaneal length has been a research hotspot for a long time and needs to be addressed^12^.

**Fig. 1:**
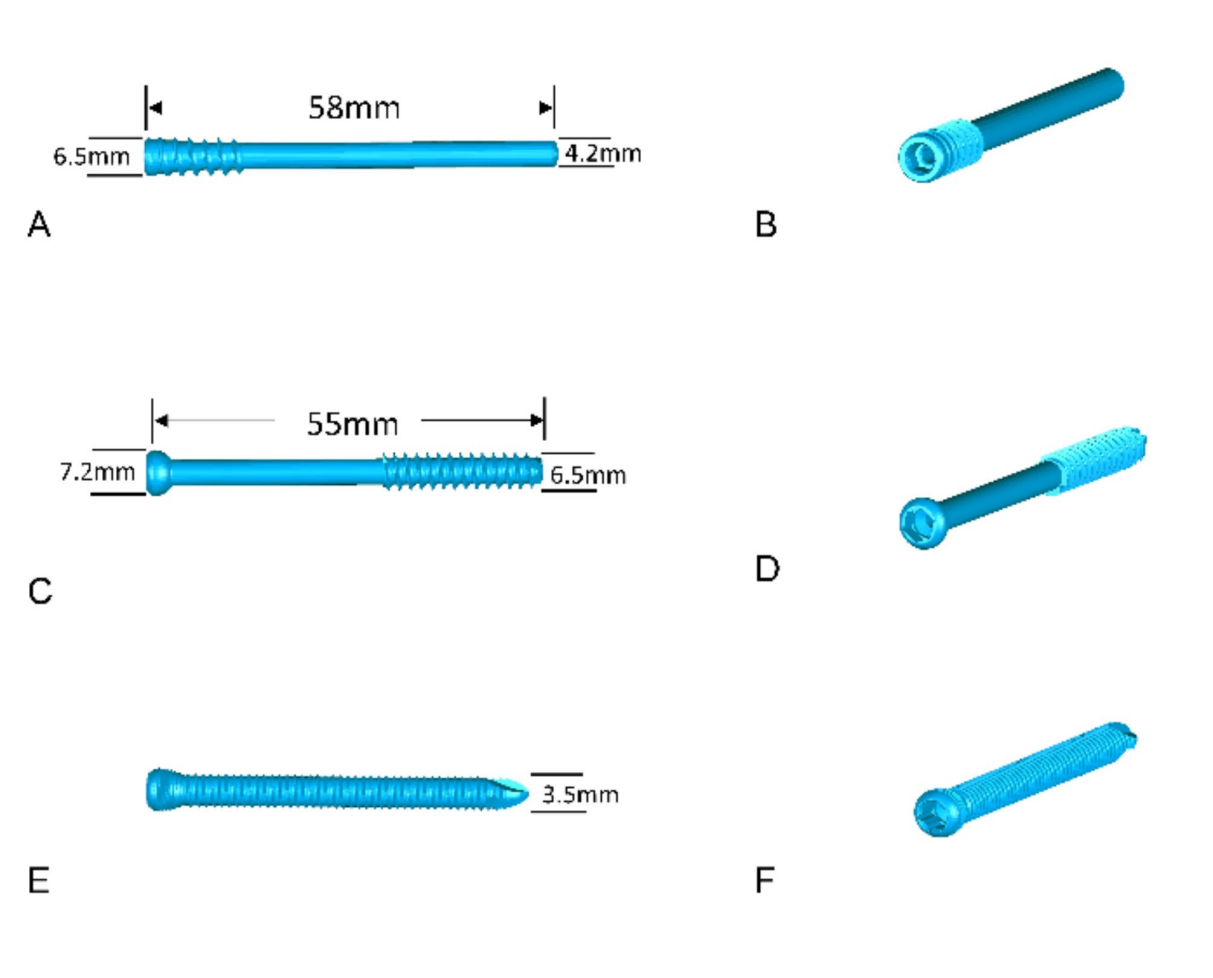
Illustration of the multiple screws. (**A**,**B**) Distracting screw. (**C**,**D**) Lag screw. (**E**,**F**) Locking screw.

Lag screws press the broken end strongly to achieve stability; however, this leads to varying degrees of calcaneal shortening deformity and is in pronounced conflict with intraoperative distraction reduction for length restoration^13^. Different locking plates with angular stability have been widely used during open reduction of calcaneal fractures^6,14–17^. A new distraction screw (Chinese patent number ZL 2017 2 0336902. 1) with threads on its proximal end was designed. This screw can offer a strong distracting force for maintaining the length. Figure 1 presents the distraction screw, lag screw, and locking screw. However, the aforementioned equipment has not been compared in terms of biomechanical parameters affecting the maintenance of calcaneal length.

By conducting computed tomography (CT)-based finite element analysis (FEA), a widely accepted virtual analysis scheme, a detailed quantitative estimation of displacement and load distributions in surgical implants and surrounding bones can be made^18^. The present study compared the distraction effect and biomechanical parameters (comprehensive displacement, stress distribution) of multiple fixation schemes for calcaneal fractures through FEA. We hypothesized that the distraction effect of distraction screws in vitro can be repeated in calcaneal body fractures in vivo.

## Materials and methods

### Patient information

A 29-year-old healthy female volunteer (height: 177 cm; weight: 72 kg) was recruited. The participant had no tumor, congenital malformation, immune system disease, or history of lower limb fracture. The present study was conducted according to the Helsinki Declaration and was approved by the Ethics Committee of the Affiliated People’s Hospital of Jiangsu University (code: K-20200148-Y). The volunteer provided written informed consent.

### Establishment of the calcaneus model

**Fig. 2:**
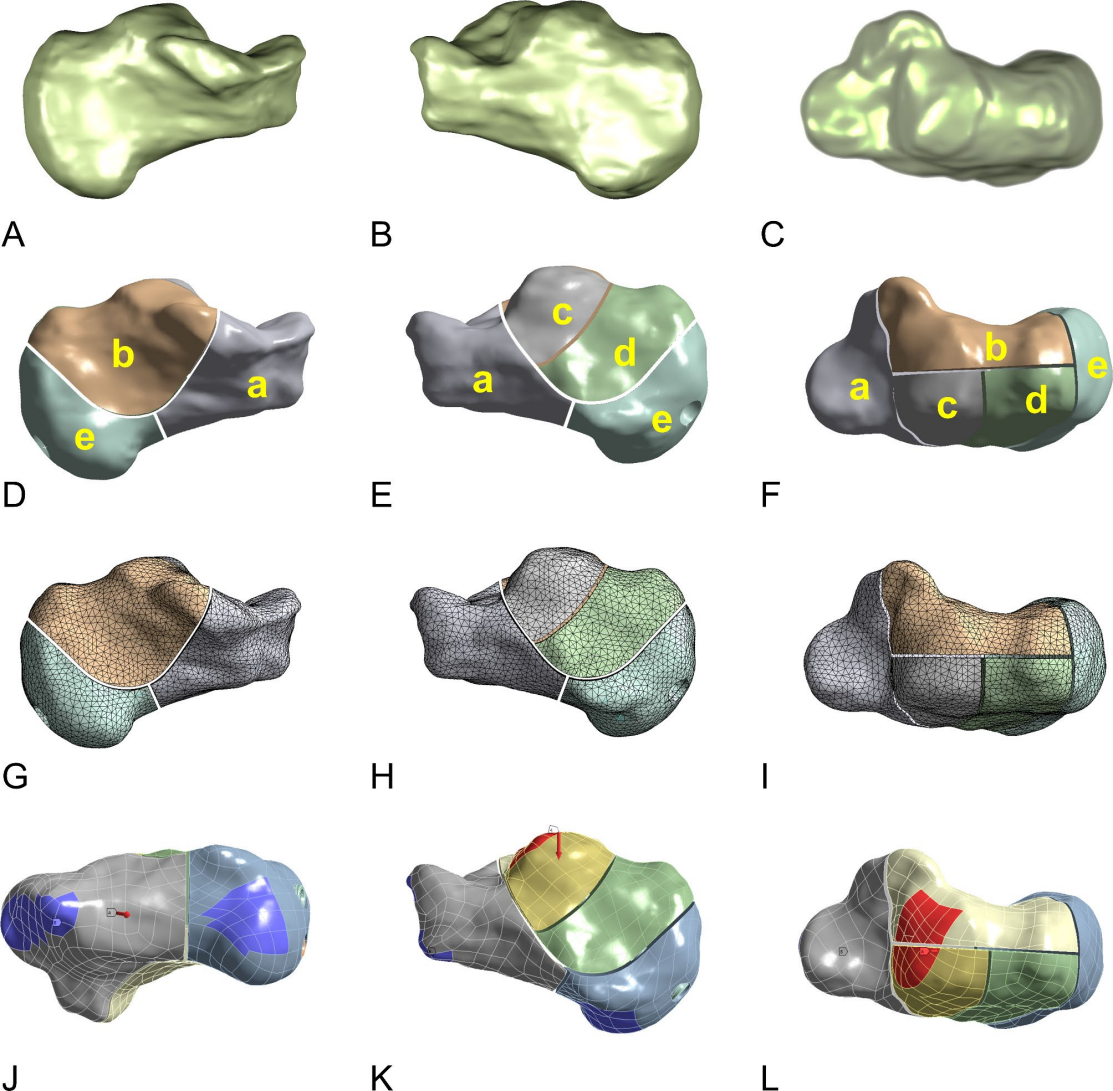
Illustractions of calcaneus model. (**A**–**C**) A model of calcaneus after smoothing. (**D**–**F**) Sanders II calcaneal fracture after cutting. (**G**–**I**) Geometric model after meshing. (**J**–**L**) Constraint point and pressure point.

**Fig. 3:**
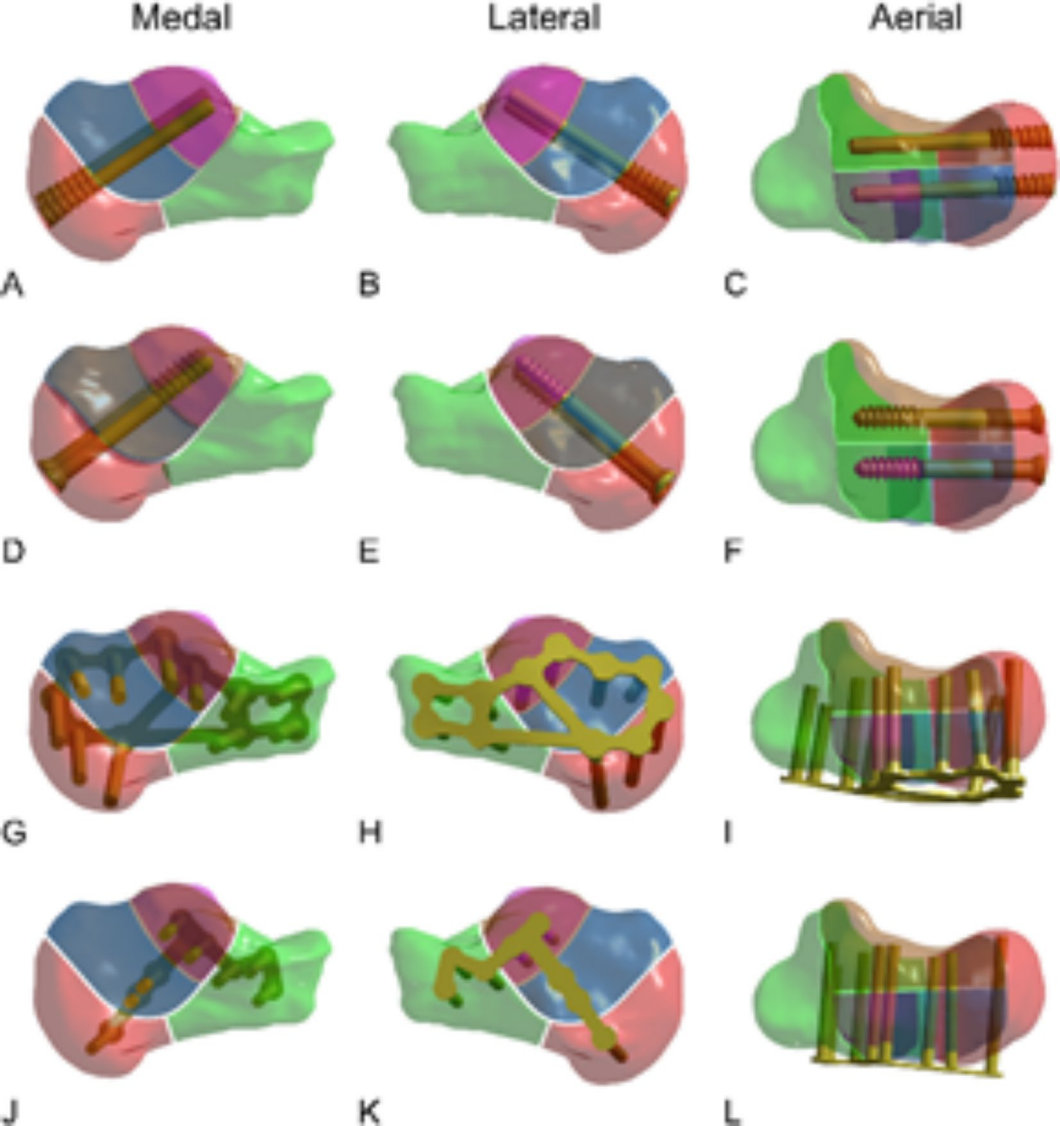
Multiple internal fixation methods and a model of calcaneal fracture. (**A**–**C**) Distracting screw. (**D**–**F**) Lag screw. (**G**–**I**) Frame-locking plate. (**J**–**L**) T-shape locking plate.

A 256-row spiral CT scanner (Philips, Netherlands) was run from the distal tibia to the distal calcaneus. The scanning slice thickness was 1.5 mm. The tube current and voltage were 500 mA and 120 kV, respectively. Then, complete calcaneal data were imported into the medical image processing software MIMICS21.0 (Materialise, Belgium) in the DICOM format. A finite element geometric model of the calcaneus was constructed, and the 3D solid model was smoothened (Fig. 2A–C). By using Boolean calculation, the calcaneal model and four internal fixation models were assembled. The generated model was processed through denoising, smoothing, and surface fitting (Fig. 3A–L).

### Establishment of the bone plate model

The calcaneal model was imported into SOLIDWORDS software (Dassault Systems, USA), and the distraction screw, semi-threaded cancellous bone screw, frame locking plate, and T-shaped locking plate were drawn according to the physical size (Double Medical, China) at 1:1.

Two screws, each having a length of 58 mm and a diameter of the threaded part of 6.5 mm, were used in the distraction screw group. The lag screw group used 2 screws, each of length 55 mm and diameter of threaded part 6.5 mm. In the frame locking plate group, 1 frame locking plate and 11 screws (diameter: 3.5 mm) were used. The T-shaped locking plate group used 1 T-shaped locking plate and 8 screws (diameter: 3.5 mm).

The calcaneal model was cut to form the Sanders II calcaneal fracture^19–21^ and primarily includes the anterior inferior fragment (a), superior medial fragment (b), superior lateral fragment (c), lateral wall fragment (d), and posterior fragment (e). A 0.5-mm cortical defect was noted between each fragment (Fig. 2D–F).

The calcaneal model and four internal fixation models were assembled using Boolean calculation. The established model was processed through denoising, smoothing, and surface fitting (Fig. 3A–L).

### Preprocessing of finite element modeling

**Fig. 4:**
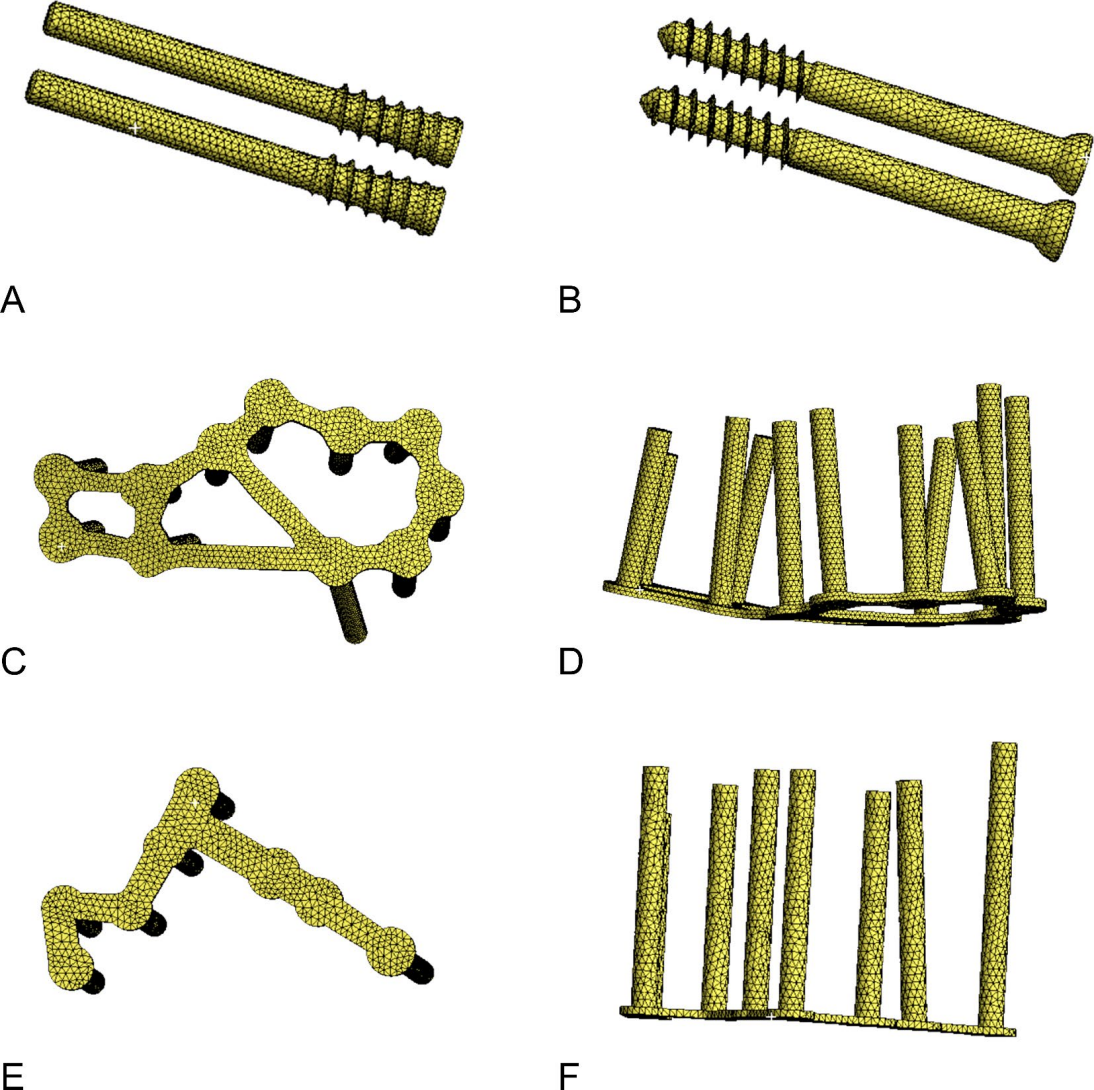
Mesh of the screws and plates. (**A**) Distracting screw. (**B**) Lag screw. (**C**,**D**) Frame-locking plate. (**E**,**F**) T-shape locking plate.

The generated four model groups were imported into ANSYS2017 (ANSYS, USA), and the mesh density was established (Figs. 2G–I, 4). The mesh was tetrahedron and its size for screws and bone was 1 and 2 mm, respectively. For the threaded portion, the mesh size ranged from 0.1 to 1 mm. Table 1 lists the number of elements and nodes. To simplify screw–bone interfaces, bonding features were assigned to simulate complete interface fixation. The threaded part of the screw was bound to the fragment. The contact between the unthreaded part of the screw and the fragment between the fragments was defined as a hard contact with friction. The friction coefficient was defined as 0.2. The contact behavior between the bone and plate was frictionless. The density of the bone cortex and cancellous bone was calculated as follows: ρ = 1.067 × HU + 131 kg/m^3^. The screws and plates were of titanium alloy and set to a homogeneous material. Table 2 presents the density and Poisson’s ratio.

**Table 1:** The number of elements and nodes in four types of internal fixation.

		Group			
		Lag screw	Distraction screw	Frame locking plate	T-shaped locking plate
All fragments	Nodes	126,231	128,984	120,008	120,406
	Elements	83,692	85,643	79,195	79,924
Fragment a	Nodes	32,621	32,724	32,893	32,664
	Elements	21,983	22,072	21,722	21,687
Fragment b	Nodes	29,113	26,955	26,364	26,694
	Elements	19,139	17,847	17,458	17,726
Fragment c	Nodes	13,983	11,440	11,349	11,283
	Elements	9053	7456	7341	7318
Fragment d	Nodes	18,934	19,028	18,738	19,059
	Elements	12,499	12,594	12,404	12,598
Fragment e	Nodes	31,580	38,837	30,664	30,706
	Elements	21,018	25,674	20,270	20,595
Implants	Nodes	Outside: 16,861	Outside: 18,661	82,756	42,102
		Inside: 16,943	Inside: 18,674		
	Elements	Outside: 10,205	Outside: 10,782	49,581	24,687
		Inside: 10,252	Inside: 10,789

**Table 2:** The density value and Poisson’s ratio.

	Calcaneus	Plate
Density value	ρ = 1.067 × HU + 131 kg/m^3^	ρ = 4650.0001 kg/m^3^
Poisson’s ratio	μ = 0.3	μ = 0.33

The tuberculum anterior calcanei, processus medialis, and lateralis tuberis calcanei were completely constrained. To assess the biomechanical responses of the multiple fixation schemes, 350, 700, and 1400 N loads were vertically exerted at the upper articular surface of the calcaneus, thus simulating biped standing, monopod standing, and jumping postures, respectively (Fig. 2J–L).

**Fig. 5:**
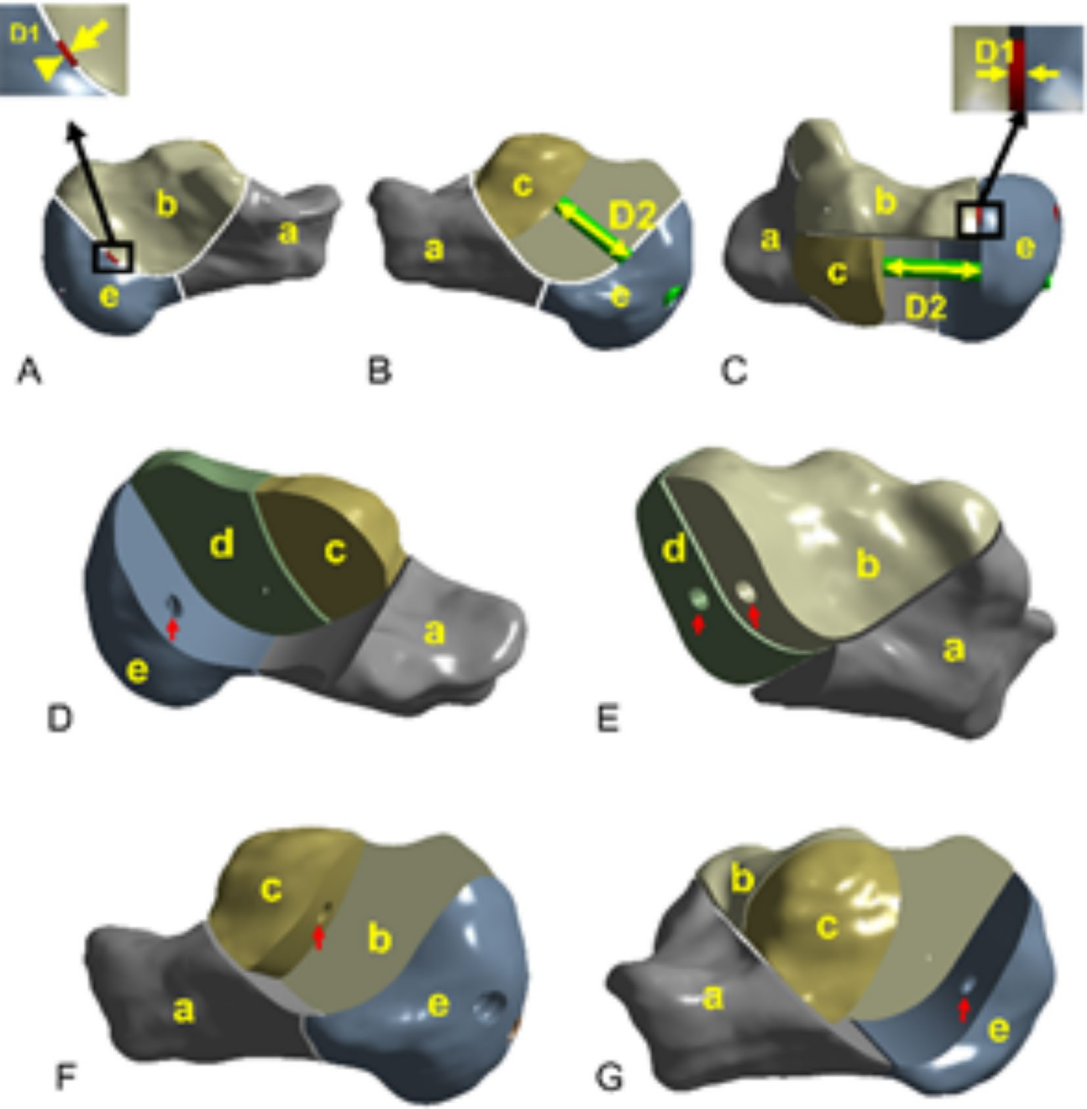
Calculation model diagram of medial and lateral shortening distances. (**A**) The medial shortening distance (D1) between the medial bone fragments. (**B**) The lateral shortening distance D2 between the lateral bone fragments. (**C**) The shortening distances D1 and D2 between the medial and lateral bone fragments. (**D**–**G**) Rings in the fragments.

In the calcaneal model, the intersection of the medial screw verse fragments (fragments b and e) resulted in two medial rings. The medial shortening distance (D1) between the center points of the two rings was calculated in the medial screw orientation (Fig. 5A, C). The intersection of the lateral screw verse fragments (fragments c and e) resulted in two lateral rings. The lateral shortening distance (D2) between the center points of the two rings was calculated in the lateral screw orientation (Fig. 5B,C). The rings were marked with red arrows (Fig. 5D–G). The same set of the three-dimensional coordinates of the rings was employed in the four models. D1, D2, von Mises stresses, and von Mises stress distribution were calculated and depicted.

### Statistical analysis

SPSS26 (IBM, USA) was used to compare the comprehensive displacement of the four model groups. Graphpad was used to draw histograms.

## Results

### Shortening distances

With the application of vertical axial stresses of 350 N/700 N/1400 N to the subtalar joint, the indices of each group gradually increased.

**Fig. 6:**
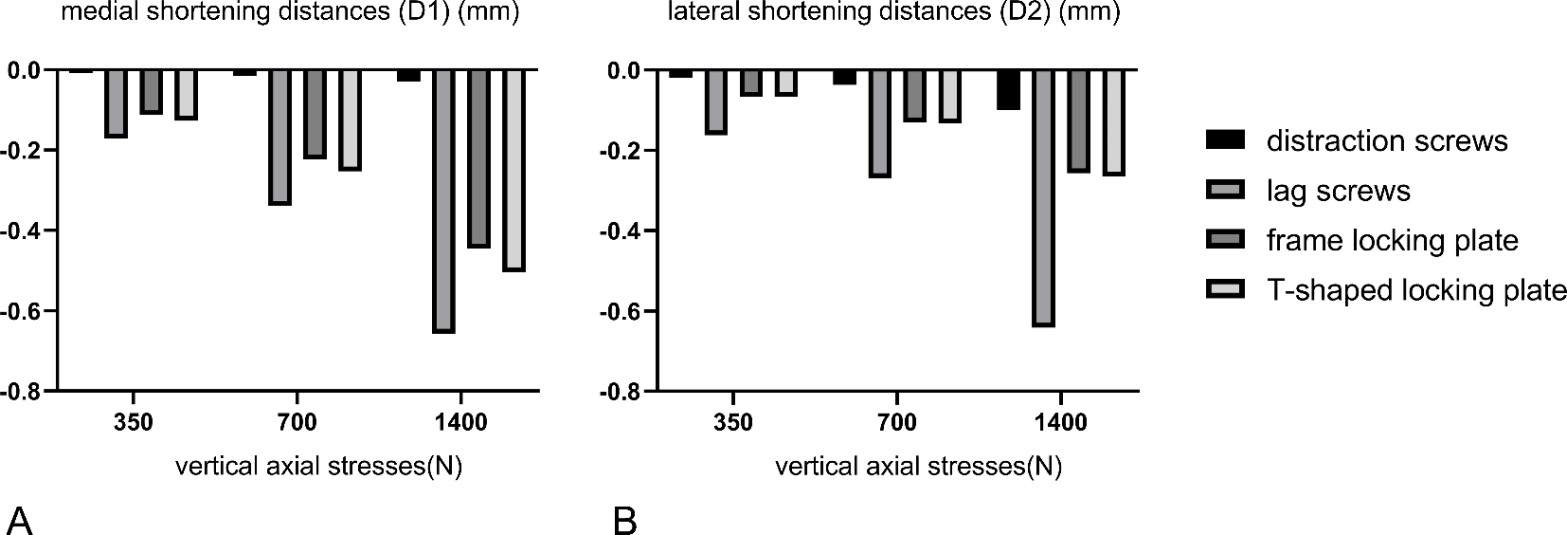
Medial (D1) and lateral (D2) shortening distances of four internal fixation schemes.

The medial (D1) and lateral (D2) shortening distances of the automatic distracting screw group were 0.008 mm/0.015 mm/0.029 mm and 0.020 mm/0.037 mm/0.100 mm for the three vertical stresses applied, respectively. The medial (D1) and lateral (D2) shortening distances of the semi-threaded cancellous bone screw group were 0.171 mm/0.338 mm/0.657 mm and 0.162 mm/0.269 mm/0.642 mm for the three loads applied, respectively. The medial (D1) and lateral (D2) shortening distances of the large frame locking plate group were 0.127 mm/0.224 mm/0.445 mm and 0.067 mm/0.130 mm/0.257 mm for the three loads applied, respectively. The medial (D1) and lateral (2) shortening distances of the T-shaped locking plate group was 0.127 mm/0.253 mm/0.504 mm and 0.067 mm/0.133 mm/0.265 mm for the three loads applied, respectively (Table 3) (Fig. 6).

**Table 3:** Medial (D1) and lateral (D2) shortening distances of four internal fixation schemes.

	Simulating biped standing (350 N)		Monopod standing (700 N)		Jumping posture (1400 N)	
	Medial (D1)	Lateral (D2)	Medial	Lateral	Medial	Lateral
Distraction screws	− 0.008	− 0.020	− 0.015	− 0.037	− 0.029	− 0.100
Lag screws	− 0.171	− 0.162	− 0.338	− 0.269	− 0.657	− 0.642
Frame locking plate	− 0.113	− 0.066	− 0.224	− 0.130	− 0.445	− 0.257
T-shaped locking plate	− 0.127	− 0.067	− 0.253	− 0.133	− 0.504	− 0.265

The overall shortening distances of the four internal fixation schemes were as follows: distraction screw group < frame locking plate group < T-shaped locking plate group < lag screw group. D2 was greater than D1 in the distraction screw group, whereas it was the other way around in the other three internal fixation methods.

### Peak von Mises stresses

**Fig. 7:**
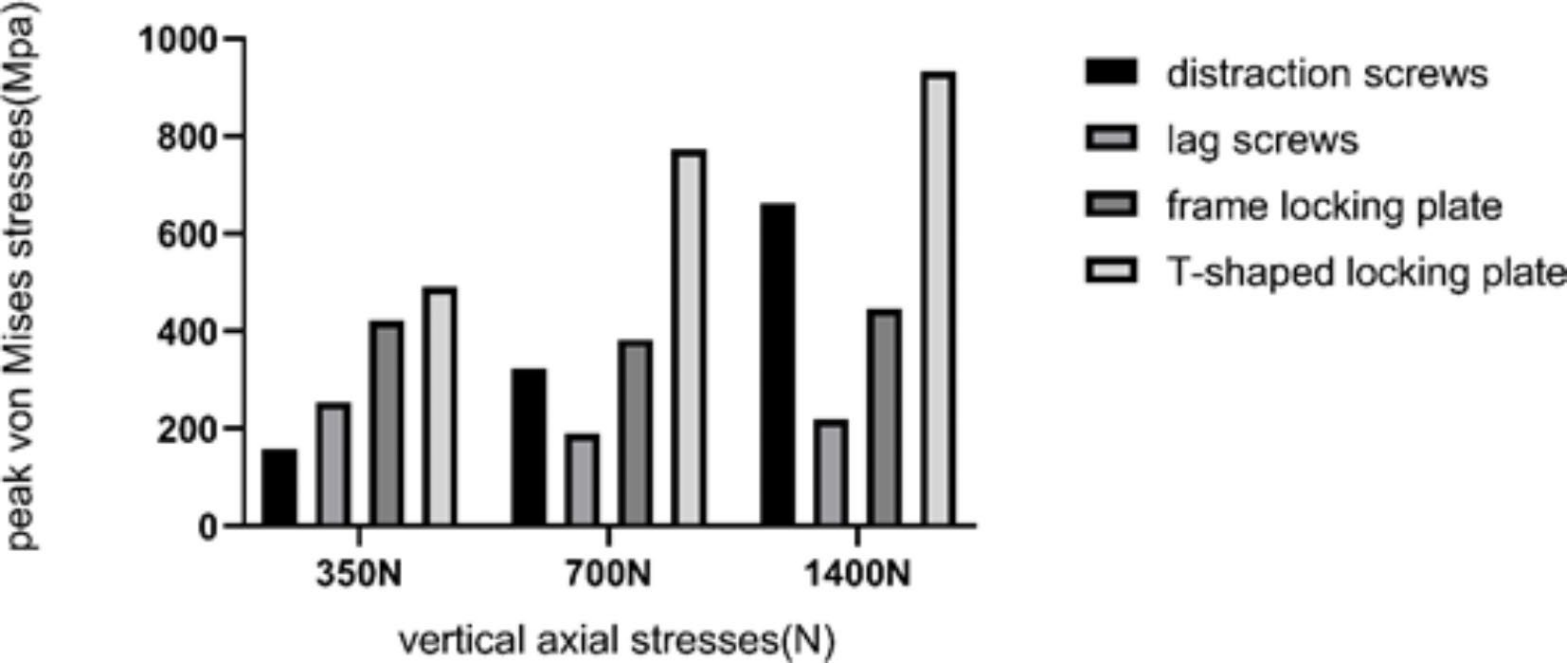
The peak von Mises stresses of four internal fixation schemes.

Following the application of 350 N/700 N/1400 N vertical axial stresses to the subtalar joint, the peak von Mises stresses of the automatic distracting screw were 158.9 MPa/323.6 MPa/663.1 MPa, respectively. Following the application of the same vertical axial stresses, the maximal von Mises stresses of the lag screw, large frame locking plate, and T-shaped locking plate were 254.8 MPa/421.4 MPa/491.0 MPa, 189.7 MPa/383.1 MPa/772.7 MPa, and 218.9 MPa/446.1 MPa/931.8 MPa, respectively (Table 4) (Fig. 7).

**Table 4:** The peak von Mises stresses of four internal fixation schemes.

	Simulating biped standing (350 N)	Monopod standing (700 N)	Jumping posture (1400 N)
Distraction screws	158.90	323.63	663.07
Lag screws	254.78	421.36	491.04
Frame locking plate	189.74	383.08	772.72
T-shaped locking plate	218.88	446.12	931.84

### Peak von Mises equivalent stresses

**Fig. 8:**
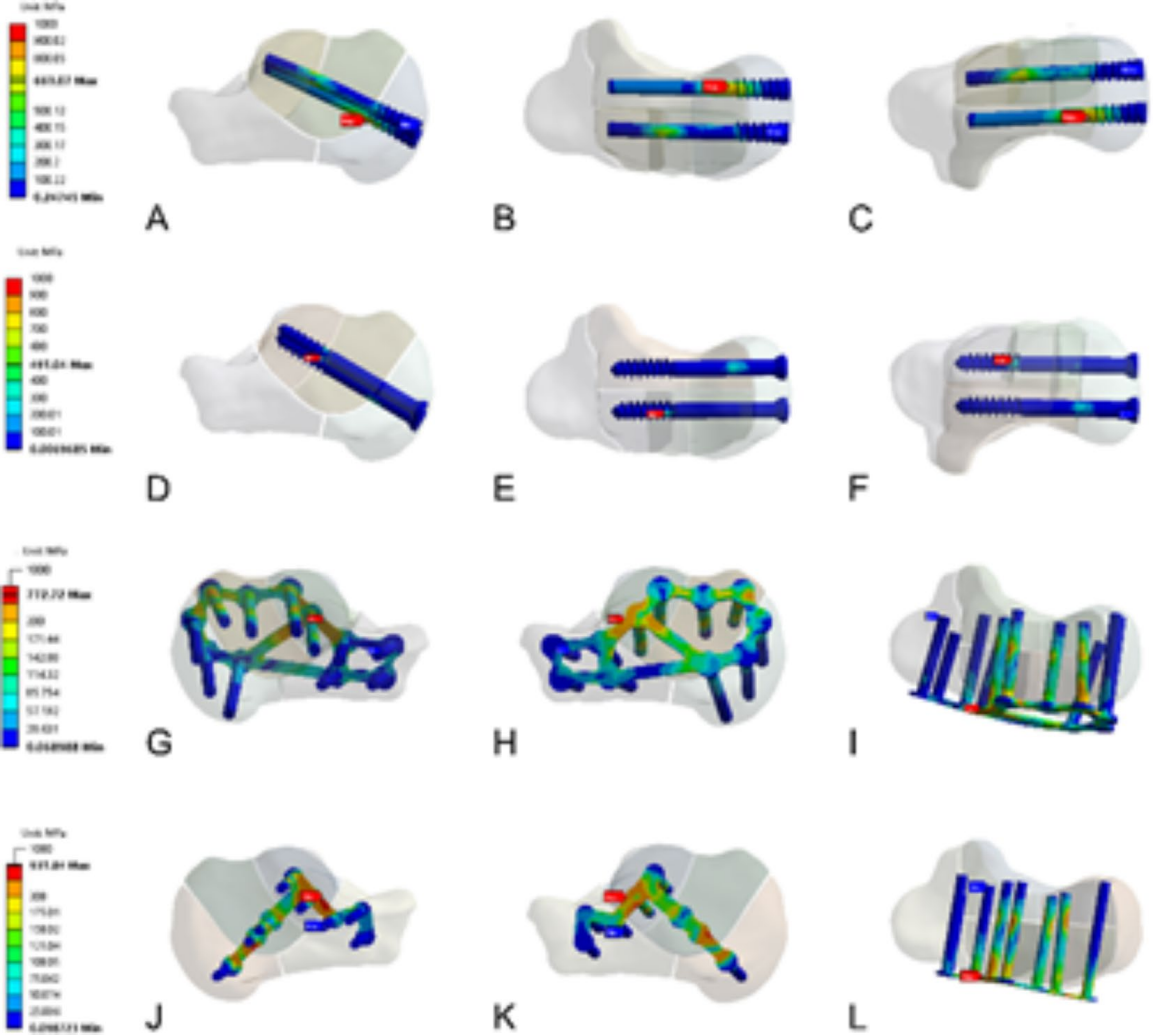
Stress distribution of internal plants in various internal fixation models. (**A**–**C**) The Peak Von Mises equivalent stress of the distraction screw group located at the junction of the thread and the screw of the inner screw. (**D**–**F**) The Peak Von Mises equivalent stress of the lag screw group located at the junction of the thread and the screw of the outer screw. (**G**–**I**) The Peak Von Mises equivalent stress of the frame locking-plate group located at the joint of the screw and plate on the outside of the fragment c. (**J**–**L**) The Peak Von Mises equivalent stress of T-shape locking-plate group located at the joint of screw and plate on the outside of fragment c.

In the distraction screw group, the peak von Mises equivalent stress was located at the junction connecting the thread and medial screw (Fig. 8A–C), while in the lag screw group, it was located at the junction connecting the thread and lateral screw (Fig. 8D–F). The peak von Mises equivalent stresses of the frame and T-shaped locking plate groups were located at the joint of the screw and plate on the outside of fragment c (Fig. 8G–L).

## Discussion

We here remodeled a calcaneal body fracture and compared the mechanical effects of various internal fixation devices on the treatment of this fracture. This is the first study reporting on calcaneal length by conducting FEA. In the presence of different loads, the forces of the calcaneus and implants under biped standing, monopod standing, and jumping postures were simulated.

The displacement degree of the fragment and the peak Von Mises equivalent stress gradually increased with an increase in the load. This is consistent with previous study results^12,22–24^. In this study, the overall shortening distances of the four internal fixation groups were as follows: distraction screw group < frame locking plate group < T-shaped locking plate group < lag screw group. Among the screws and the locking plates, the distraction screw was the best in resisting shortening deformities and maintaining calcaneal length. This finding is of great clinical significance.

The distraction screw changes the screw thread to the back half of the screw, which is an innovative design. By contrast, the lag screw changes the screw thread to the front half of the screw. These two screws thus exert opposite mechanical effects because they have completely different designs. During distraction screw implantation, the first part is a round, unthreaded rod. On screwing in the threaded part, this part cuts the posterior fragment, thereby producing a strong stretching force and pushing the two fragments apart. According to the result, the internal fixation device with the distraction effect rather than the compression effect seems helpful in maintaining calcaneal length in the comminuted calcaneal body fracture.

Unlike distraction screws, the extensively used lag screws can produce a strong compression effect between large fragments, which aggravates shortening deformities in the comminuted calcaneal body fracture. The two locking plates exhibit a better effect than a lag screw because the lag screw exerts a compression effect. In a previous FEA, the efficacies of a screw and plate in calcaneal fracture treatment were compared^12,22,23,25^. The results revealed that the screw and plate had similar biomechanical stability. However, previous studies have not focused on the change in calcaneal length^12^. The present study found that calcaneal shortening deformities are obvious when lag screws are used. The results suggested that lag screws should be avoided in minimally invasive surgery for comminuted calcaneal fractures.

Different types of locking plates have been widely used for calcaneal fracture treatment^26,27^. The locking plate exerts no reduction effect, so a tool for calcaneal length restoration should be used before implanting the plate^28^. In this study, the distraction effects of the two types of locking plates were worse than that of the distraction screws, but better than that of the lag screws. This result may be related to their fixed pattern differences. The locking plate belongs to eccentric fixation, the distraction screw belongs to central fixation. Furthermore, the distraction screw displayed a stronger effect on calcaneal length maintenance.

Except in the distraction screw group, the medial shortening distance (D1) was greater than the lateral shortening distance (D2) in the remaining internal fixation groups. This may be because to achieve better lateral stability, the plates and screws were placed on the outside of the calcaneus. Moreover, the loads were exerted vertically at the subtalar joint, and the medial fragment occupied a larger compression area (Fig. 2L). The lateral shortening distance (D2) was greater than the medial shortening distance (D1) in the distraction screw group. The possible reason for the smaller D1 is that the medial cortex is thicker than the lateral cortex even when the medial and lateral distraction screws have the same support strength. The distraction screw exerts a strong stretching effect and allows accurate reverse injury treatment, so as to achieve restoration of the length of the original shortening deformed fragments instead.

When the 1400 N vertical axial stress was applied to the subtalar joint, the peak von Mises stresses of the distraction screw, lag screw, frame locking plate, and T-shaped locking plate were 663.07, 491.04, 772.72, and 931.84 Mpa, respectively.

The T-shaped steel locking plate had higher yield strength than the titanium alloy locally at 816.6 Mpa. The peak von Mises stress of the T-shaped locking plate was higher, possibly because screw holes were fewer and stress was more concentrated. The local stress concentration may lead to internal fixation failure. The peak von Mises stresses of the three internal fixation methods, except that of the T-shaped locking plate, were within the yield strength of the titanium alloy at 816.6 Mpa. The peak von Mises stresses of the fixation mode were lower for the two screw groups than for the two plate groups. This may be because the direction of shortening displacement between the fragments was similar to direction of the screws, and adequate shear force was lacking. The locking plates are relatively stable, with fretting occurring between the fragments of the locking plates. The plates exert a weak effect on the axial calcaneal shortening deformities.

The peak stresses of the two locking plates were located at the junction connecting the screw and locking plate. These results are similar to those of Lee and Ni^22,29^. The position of the stress concentration may be the location of failure of the internal fixation device. This suggested that the mechanical strength of the corresponding part should be increased and optimized while designing the plate.

Starting from the fixed mode, the screw compression technology belongs to the absolutely stable mode, which renders the bone mass very stable when used to fix it. The stable mode-fixed bone mass also exhibits pressurized contact. The calcaneal length originally compressed by violence becomes shorter because of the compression effect of the lag screws, which possibly leads to severe shortening deformities, with the patient even requiring revision surgery^4,30,31^.

The bone mass fixed using the automatic distraction screw exhibited no fretting on the screw’s long axis and belonged to an absolutely stable mode. A swing may occur in the lateral direction perpendicular to the long axis because the bone mass is stretched out. Including lateral tension, screws can prevent this swing. Locking plate fixation is a relatively stable internal fixation method^16,27^, and fretting occurs between the broken ends of the bone mass. Larger incisions are associated with greater wound nonunion and infection risks^23^. Locking plates are expensive. A semi-threaded screw used as a lag screw functions similar to a continuous pressure reduction clamp/compression device in the body. The locking plate can maintain the existing fixed shape and resist shear force^32^. The newly designed automatic distracting screw functions similarly to a continuous tension reduction forceps/reduction device in the retention body.

Distraction screws offer a strong distraction force between fragments, an innovation, and supplement in the orthopedic internal fixation field. In addition to fresh calcaneal body fractures, distraction screws can be used for calcaneal defects ^4^, flatfoot^33^, and other operations requiring an increase in calcaneal length. Moreover, distraction screws can be used for fractures wherever length restoration (e.g., for tibial plateau fractures, proximal humerus fractures, and pilon fractures).

### Limitations

The study has some limitations. First, the model discussed in the present study is our simulation model. We developed it based on common clinical calcaneal fractures and by referring to the article on three-dimensional fracture line models of the calcaneus, which has not yet received widespread validation. Second, fixation methods such as full thread screws, double head screws, a locking plate + screw combination, and an external fixator were not considered. Third, we only investigated calcaneal body fractures were investigated, and other types of calcaneal fractures were not considered. The effects of muscle, ligament, and fascia were ignored in the computer simulation step. The experimental results must be further confirmed through biomechanical and clinical studies.

## Conclusion

In patients with calcaneal body fractures, the distraction screw is a potential therapeutic option for resisting calcaneal shortening.

## Data Availability

No datasets were generated or analysed during the current study.
