# Author Correction: Finite element analysis of restoring length with multiple internal fixations in calcaneal body fracture

**DOI:** 10.1038/s41598-025-89120-y

**Published:** 2025-02-07

**Authors:** Xiang Yao, Peiqi Ding, Chong Wang, Han Miao, Yicong Chao, Jiawei Wang, Minjie Hu, Jilei Tang

**Affiliations:** 1 Department of Orthopaedics, The Affiliated People’s Hospital of Jiangsu University, Zhenjiang, 212000 Jiangsu China; 2 Jiangsu University, Zhenjiang, 212000 Jiangsu China; 3 Department of Orthopaedics, The People’s Hospital of Danyang, Zhenjiang, 212300 Jiangsu China; 4 Department of Orthopaedics, Qidong Hospital of Traditional Chinese Medicine, Nantong, 226200 Jiangsu China

Correction to: *Scientific Reports* 10.1038/s41598-024-75267-7, published online 10 October 2024

The Funding section in the original version of this Article was omitted.

The Funding section now reads: “Clinical Medicine Science and Technology Development Foundation of Jiangsu University (JLY2021002). Medical Education Collaborative Innovation Fund of Jiangsu University (JDYY2023013). Special fund of Science and Technology Program of Jiangsu Province (BE2023756)”.

The original Article has been corrected.

